# Effects of autoclaving and high pressure on allergenicity of hazelnut proteins

**DOI:** 10.1186/2043-9113-2-12

**Published:** 2012-05-22

**Authors:** Elena López, Carmen Cuadrado, Carmen Burbano, Maria Aranzazu Jiménez, Julia Rodríguez, Jesús F Crespo

**Affiliations:** 1 Centro de Investigación (i + 12) del Hospital Universitario 12 de Octubre, Avda de Córdoba, s/n, 28041, Madrid, Spain; 2 Departamento de Tecnología de Alimentos, SGIT-INIA, Ctra. La Coruña Km 7.5, 28040, Madrid, Spain; 3 Servicio de Alergia del Hospital Universitario 12 de Octubre, Av. de Córdoba s/n, 28040, Madrid, Spain

**Keywords:** Structural analysis of allergen-proteins and Glycosylation

## Abstract

**Background:**

Hazelnut is reported as a causative agent of allergic reactions. However it is also an edible nut with health benefits. The allergenic characteristics of hazelnut-samples after autoclaving (AC) and high-pressure (HHP) processing have been studied and are also presented here. Previous studies demonstrated that AC treatments were responsible for structural transformation of protein structure motifs. Thus, structural analyses of allergen proteins from hazelnut were carried out to observe what is occurring in relation to the specific-IgE recognition of the related allergenic proteins. The aims of this work are to evaluate the effect of AC and HHP processing on hazelnut *in vitro* allergenicity using human-sera and to analyse the complexity of hazelnut allergen-protein structures.

**Methods:**

Hazelnut-samples were subjected to AC and HHP processing. The specific IgE- reactivity was studied in 15 allergic clinic-patients via western blotting analyses. A series of homology-based-bioinformatics 3D-models (Cora 1, Cora 8, Cora 9 and Cora 11) were generated for the antigens included in the study to analyse the co mplexity of their protein structure. This study is supported by the Declaration of Helsinki and subsequent ethical guidelines.

**Results:**

A severe reduction *in vitro* in allergenicity to hazelnut after AC processing was observed in the allergic clinic-patients studied. The specific-IgE binding of some of the described immunoreactive hazelnut protein-bands: Cora 1 ~18KDa, Cora 8 ~9KDa, Cora 9 ~35-40KDa and Cora 11 ~47-48 KDa decreases. Furthermore a relevant glycosylation was assigned and visualized via structural analysis of proteins (3D-modelling) for the first time in the protein-allergen Cora 11 showing a new role which could open a new door for allergenicity-unravellings.

**Conclusion:**

Hazelnut allergenicity-studies *in vivo* via Prick-Prick and other means using AC processing are crucial to verify the data we observed via *in vitro* analyses. Glycosylation studies provided us with clues to elucidate, in the near future, mechanisms of the structures that contribute to hazelnut allergenicity, which thus, in turn, help alleviate food allergens.

## Introduction

Food allergies are receiving more attention and seem to be increasing, especially in Western countries [1-5]. Consumers are becoming more aware and are more informed about food sensitivities, including allergies. Allergies to hazelnuts, tend to be of a more severe nature, causing life-threatening and sometimes fatal reactions. Indeed, symptoms upon hazelnut- ingestion are often confined to the mouth and throat, but severe systemic reactions have been described in some patients [6-14]. Also, hazelnut is a good habit in a healthy nutritional regimen. Type I food allergy is defined as an IgE-mediated response to a protein (or proteins) in a food source. It is not known why a food protein that is innocuous and well-tolerated by most individuals, triggers an allergic response in sensitive individuals [15-17].

The portion of the food protein recognized by IgE is called the epitope. Epitopes are generally categorized as linear or conformational, where linear epitope involves a contiguous stretch of amino acids, and a conformational epitope involves non contiguous amino acids which form a three-dimensional/structural motif. Individual patients may differ significantly in their sensitivity toward an allergen; however, the basis of such differential sensitivities remains to be elucidated [17,18].

Moreover, studies of protein thermal conformational stability under different treatments from both biochemical and techno functional points of view need to be extended [19,20]. In addition, it has been suggested that modifications in allergenicity caused by thermal treatments could provide a better understanding of the risk of introducing hazelnuts into human daily food intake. Furthermore Hansen *et al.,*[21] indicated that roasting of hazelnuts reduces the allergenicity. Also, Ortolani *et al.,*[22] selected subjects with a history of allergic reactions on ingestion of hazelnut and determined how many had true allergy by means of the double-blind, placebo-controlled food challenge (DBPCFC), showing that 78% of the analyzed subjects had a positive DBPCFC result.

In fact, allergy to hazelnuts has been frequently reported, but data on the identification and characterization of the hazelnut allergens, as well as on the reliability of the methods for *in vitro* and *in vivo* detection of specific IgE for these allergens, are scarce. In addition, different researches have indicated that post-translational modifications (PTMs) like phosphorylation and glycosylation can play a relevant role in allergenicity related to edible nuts including hazelnuts [23]. On the other hand, sequence and structural information improve the prediction of allergenic peptides. Motifs can also be mapped onto the 3D-structure of a protein to identify epitopes and conserved functional areas [24]. Combining sequence analysis with structural representations can answer many questions about the nature of the IgE epitopes of allergens [25]. While single amino acid differences may be quite important in individual reactivity, a 3D view of the identified IgE binding sites can provide missing information about the possible relationships between structure and sequence [26].

We selected the hazelnut as an edible nut for its health benefits which makes the hazelnut food processing industry one of significant economic impact and profit [27-29]. The present study was specifically designed to evaluate the effects of AC and HHP processing on the allergenic characteristics of proteins from hazelnuts. This study aimed to combine functional research protein-allergen analysis via western blotting and structural analysis of allergen proteins from the hazelnut, in order to observe what is occurring in relation to the specific recognition of the allergenic proteins from hazelnuts by IgE using sera from patients allergic to hazelnuts with clinical significance. In addition, and in order to analyze the complexity level of the structural arrangements of the studied allergens, a bioinformatics study was done, generating 3D structural models for all of them looking for putative glycosylation motifs. As a general result, in all cases the structural complexity of the modelled proteins could act as a molecular explanation for the effects observed after AC treatment. As was previously demonstrated [30], AC treatment significantly modifies the proportion of secondary structural elements in proteins, being thus putatively responsible for their changes in allergenicity.

## Materials and methods

### Statement of ethical approval

This study was conducted in compliance with the international “Declaration of Helsinki.” An informed consent about the procedures as well as permission from the Ethical Committee of 12 de Octubre and Carlos III Hospitals of Health was obtained (http://www.madrid.org/cs/Satelite?pagename=Hospital12Octure/Page/H12O_home, http://www.madrid.org/cs/Satellite?pagename=HospialCarlosIII).

This study adhered to the tenets of the Declaration of Helsinki. (http://www.wma.net/e/policy/b3.htm). (Declaration of Helsinki (1964), Belmont (1978) and agreement of Oviedo (1997) - the basic principles for human and biological samples research studies -) (http://www.isciii.es/htdocs/index.jsp). (“Working link”: http://www.madrid.org/cs/Satelite?pagename=Hospital12Octure/Page/H12O_home).

### Plant material, AC and HHP processing

Raw and processed seeds were milled to pass through a 1 mm sieve (Tecator, Cyclotec 1093, Hoganas, Sweden), and the resulting meal was defatted with n-hexane (34 mL/g of flour) for 4 h, shaken, and air-dried after filtration of the n-hexane. Defatted flour was extracted twice in a solution of Sodium borate, pH 8.0, plus 0.5 M NaCl at a 1:10 w/v ratio for 1 h at 4°C by stirring. The extract was clarified by centrifugation at 27000 g for 20 min at 4°C, and the supernatants were dialyzed against H2O for 48 h at 4°C using a dialysis membrane with a cut-off of 3.5 kDa and freeze-dried. The protein content of each sample was measured according to the Leco dye-binding assay (Bio-Rad, Hercules, CA) using bovine serum albumin (BSA; Sigma, St. Louis, MO) as a standard. Defatted hazelnut flour samples (30 g) were hydrolyzed (water 1:4 w/v, for 20 h, at 4°C). The obtained solutions were subjected to centrifugation (1200 rpm, 10 min.) and different processing of AC and HHP were applied. The resulting pellets and supernatants were frozen and stored at -20°C. The AC and HHP processing applied were: 121°C 15 min., 121°C 30 min., 138°C 15 min., 138°C 30 min. and 300Mba, 400Mba, 500Mba and 600Mba, respectively. All the processed hazelnut flour samples were centrifuged and frozen in order to obtain the lyophilized ones to be analyzed. (Note: Mba: units Milibar pressure, AC: Autoclave heat process, HHP: high pressure process).

### SDS-PAGE gel and immunoblotting analyses

Denaturing protein and SDS-PAGE gel were performed essentially according to the method of Fling and Gregerson [31]. Samples (10 g per well) were mixed 5:1 with loading buffer (8% SDS, 8 mM EDTA, 40% glycerol, 1 M -mercaptoethanol (*-*ME), and 0.01% bromophenol blue in 0.25 M Tris–HCl, pH 7.5) heated at 100°C for 10 min, electrophoresis in 4–20% gradient analytical SDS-polyacrylamide gels employing a Mini-Protean III apparatus (Bio- Rad), and either stained with Coomassie Brilliant Blue R-250 or transferred to poly (vinylidene difluoride) (PVDF) membranes (Bio -Rad). For immunoblot, proteins (25 μg) were electrophoretically transferred from the gels to PVDF by applying a constant current of 250 mA during 1.30 h at room temperature, essentially according to the method of Towbin *et al.*[32]. The membranes were stained with red-ponceau to verify protein transfer. Western immunoblotting using pooled human sera was done, as described later, with the necessary modifications to optimize the hazelnut sample analysis for this study [33]. Blots were blocked in phosphate-buffered saline plus 0.1% Tween 20 containing 1% fat-free milk powder for 2 h at room temperature and then incubated overnight at room temperature with individual and pooled sera (1:5 dilution). For individual serum and also for the pool of sera, a mouse anti-human IgE mAb HE-2 ascitic fluid was used as first antibody (diluted 1:4000 in blocking buffer) for 1 h at room temperature (RT). After 4 washing steps (10 mins each one with phosphate-buffered saline plus 0.1% Tween 20), a goat anti-mouse IgG peroxidise- conjugated antibody was used as second antibody (diluted 1:5000 in ½ v/v in blocking solution) for 1 h at RT. Finally, immunoreactive bands were visualized using the ECL chemiluminiscent kit (Bio-Rad). Coomassie-stained gels and immunostained membranes were scanned using a HPS scanjet 5590P densitometer. The files generated were analyzed with Quantity One software (Bio-Rad) using the precision plus protein standard TM (Biorad) prestained SDS-PAGE protein mixture (Bio-Rad) as standard.

### Immuno-CAP-FEIA allergy blood test

For the CAP (fluorenzymeimmunoassay CAP Pharmacia Diagnostic, Uppsala, Sweden) tests, each serum was incubated with the same volume of progressive dilutions (0.001–1 mg/mL) of each inhibitor protein solution in PBS buffer for 14 h at 4°C with agitation. After incubation, the samples were centrifuged, hazelnut commercial Immuno-CAP was then added, and specific IgE levels were assessed, according to the CAP System procedure (Pharmacia Diagnostic, Uppsala, Sweden). BSA was used as negative control. The assay was performed in duplicate samples, and each one was tested in duplicate [34-36].

### Patients

**Figure 1:**
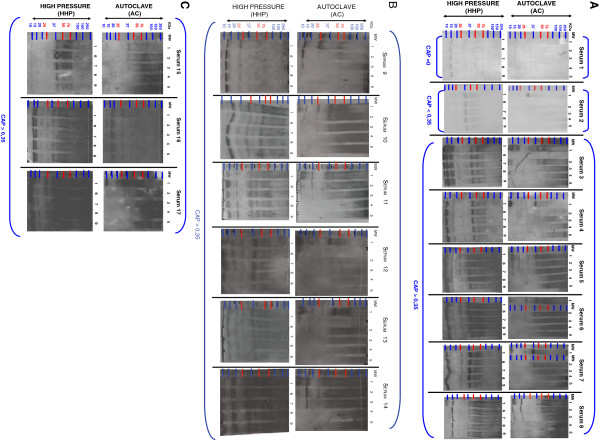
**Western blotting analysis of hazelnut processed by AC and HHP treatments.** Western blotting analysis of AC/HHP flour hazelnut samples incubated with clinical sera from patients allergic to hazelnut (*) Figure2 is illustrated in 3 sections **(A)**, **(B)** and **(C)** in order to visualize 34 western blots in a proper way. Number 1 corresponds to raw hazelnut flour; numbers 2, 3, 4 and 5 correspond to AC 121°C 15 mins., 121°C 30 mins., 138°C 15 mins., and 138°C 30 mins., respectively. Numbers 6, 7, 8 and 9 correspond to HHP 300Mba, 400Mba, 500Mba and 600Mba respectively. Sera 1 and 2 correspond to non allergic patients to hazelnut (negative controls). (Note: (i) The film-exposition - after the ECL incubation- took longer than 24 h to visualize the bands of the negative controls: sera 1 and 2) (ii) The film-exposition - after the ECL incubation- took between 5 s and 1 min. according to the different sera tested in order to visualize the bands: from serum 3 until 17) (MW: molecular weight, AC: autoclave process, HHP: high pressure process, KDa: kilodalton).

Fifteen patients (from num.3 until num.17- Figure1 A, B, C) with detectable hazelnut- specific IgE as quantified by the CAP-FEIA assay (> 0.35 kilounits/L) (Pharmacia Diagnostic, Uppsala, Sweden) (Table1), were use to evaluate the reduction in allergenicity by thermal treatment of hazelnut. The fifteen patients were selected as they belong to hazelnut-allergic patients without allergy to another edible nut.

**Table 1:** Illustration of clinic symptoms of patients allergic to hazelnut

**Patient**	**Age**	**Sex**	**Reported symptoms**	**CAP**
# 1	57	F		0
# 2	33	F		<0.35
# 3	26	M	Oral allergy syndrome	2.68
# 4	33	F	Acute urticaria	2.5
# 5	16	F	Avoidance	2.97
# 6	28	F	Oral allergy syndrome	4.03
# 7	16	F	Oral allergy syndrome	2.08
# 8	21	M	Systemic anaphylaxis	4.6
# 9	26	F	Rhinoconjunctivitis, angioedema, oral allergy syndrome	0.58
# 10	27	F	Systemic anaphylaxis	1.92
# 11	30	M	Oral allergy syndrome	2.42
# 12	42	F	Oral allergy syndrome	1.9
# 13	28	M	Avoidance	0.87
# 14	57	F	Oral allergy syndrome	2.5
# 15	47	M	Oral allergy syndrome	4.13
# 16	30	F	Avoidance	0.42
# 17	49	M	Avoidance	0.47

Patients allergic to hazelnut proteins showed clinic symptoms from oral allergy syndrome, acute urticaria, systemic anaphylaxis, rhinoconjunctivitis, angioedema to avoidance (avoidance: advised to avoid hazelnut food because of a positive skin test). The resulting data including the age and sex, are illustrated in this table. (Note: F: female, M: male).

Avoidance*: advised to avoid the food because of a positive skin test, without a clinical history of hazelnut allergy.

In addition, patient num.1 and patient num.2 –Figure1 A- were used as negative controls - with negative specific IgE recognition to hazelnuts and quantified by using the (CAP-FEIA assay (< 0.35 kilounits/L, Pharmacia Diagnostic, Uppsala, Sweden). The negative controls were selected as they belong to healthy patients without allergy to edible nuts in order to focus the data in allergen-hazelnut recognised protein-bands from human IgE hazelnut- allergic patients.

### Structural analysis and modelling procedures of hazelnut proteins Cor_a_1.04, Cor_a_8, Cor_a_9 and Cor_a_11

3D models of hazelnut alergens Cor_a_1.04 (Uniprot codes: Q9FPK2, Q9FPK3, Q9FPK4 and Q9SWR4), Cor_a_8 (Uniprot code: Q9ATH2), Cor_a_9 (Uniprot code: Q8W1C2) and Cor_a_11 (Uniprot code: Q8S4P9) were generated using homology modelling procedures and the coordinates of the following Protein Data Bank structures as templates:

Cor_a_1.04. Template: Crystal structure of major pollen allergen Bet v 1d/h from *Betula pendula*. Protein Data Bank code: 3 K78 [37]. Blast E-value: 6.1x10 − 55. Sequence Identity: 66%

Cor_a_8. Template: Crystal structure of Pru p3, non-specific lipid transfer protein from *Prunus persica*. Protein Data Bank code: 2ALG_A [38]. Blast E-value: 1.3x10 − 29. Sequence Identity: 59%

Cor_a_9. Template: Crystal structure of the hexameric form of Pru du amandin from *Prunus dulcis*. Protein Data Bank code: 3EHK [39]. Blast E-value: 1.4x10 − 146. Sequence Identity: 56%

Cor_a_11. Template: Crystal structure of soybean beta-conglycinin beta homotrimer. Protein Data bank code: 1IPK [40]. Blast E-value: 2.0x10 − 63. Sequence Identity: 354%

Models were built using the SWISS-MODEL server [41-43] available at http://swissmodel.expasy.org//SWISS-MODEL.html, and their structural quality was checked using the analysis programmes provided by the same server (Anolea/Gromos). Global model quality estimation scores QMEAN4 [44] were: Cor_a_1.04: 0.741, Cor_a_8: 0.671, Cor_a_9: 0.548 and Cor_a_11: 0.608, that are within the range of those accepted for homology-based structure models. To optimize geometries, models were energy minimized using the GROMOS 43B1 force field implemented in DeepView [45,46], using 500 steps of steepest descent minimization followed by 500 steps of conjugate-gradient minimization.

Figures were generated using the Pymol Molecular Graphics System (Schrödinger, LLC). [SWISSPROT database http://expasy.org/sprot/].

## Results

### SDS-PAGE gel and immunoblotting analyses

(a) The allergenicity was analyzed by IgE-immunoblotting. In the case of the raw- hazelnut flour (positive control), IgE antibodies from 15 individual sera allergic to hazelnut recognized bands at (a) ~ 9 KDa bands which correlate with Cor_a_8 protein –non-specific lipid transfer proteins family (nsLTPs), (b) ~ 18 KDa bands which belongs to Cor_a_1.04 protein –Bet v1 family-, (c) ~35–40 KDa bands which belong to Cor_a_9 protein – 11 S seed storage globulin, cupin family- and (d) ~47–48 KDa bands which belong to Cor_a_11 protein –Vicilin family-. A severe reduction in allergenicity to hazelnut flour *in vitro* after autoclaving at 121°C 15 mins., 121°C 30 mins., 138°C 15 mins and 138°C 30 mins, was observed in the allergic clinic patients with a CAP value > 0.35 kilounits/L by western blotting analysis. No reduction in allergenicity *in vitro* after HHP treatments was observed in any serum of the 15 patients, which were also analyzed by immunoblotting when using the same conditions for all the sera analyses [31-33].

(b) Autoclaving 121°C 15 mins and 121°C 30 mins hazelnut flour samples, which were analyzed by western blotting showed that, mainly the IgE binding of some of the described immunoreactive hazelnut protein-bands (Cor_a_1 ~18KDa, Cor_a_8 ~9KDa-, Cor_a_9 ~35–40KDa) decreases. This occurred in the majority of sera tested. Autoclaving treatments 138°C 15 mins and 138°C 30 mins of hazelnut flour samples, which were analyzed by western blotting, showed that the IgE binding of all of the described immunoreactive hazelnut protein-bands (Cor_a_1 ~18 KDa, Cor_a_8 ~ 9 KDa-, Cor_a_9 ~35-40 KDa, Cor_a _11 ~47–48 KDa) disappear. This occurred in all the sera tested (15 western blotting analyses from 15 clinical sera of 15 patients diagnosed with actual allergy to hazelnuts). [Molecular weights (MW) and allergen names are described in Database Food Allergies and Swissprot Database - ExPaSy Proteomic Server respectively; http://foodallergens.ifr.ac.uk/default.html, http://www.uniprot.org/uniprot/, http://expasy.org/sprot/].

(c) All protein bands from Coomassie-stained gel and immunostained membranes were scanned using a HPS scanjet 5590P densitometer. The files generated were analyzed with Quantity One software (Bio-Rad), which allowed us to visualize the severe reduction in allergenicity *in vitro* via the decrease of intensity of the protein-bands when comparing the raw-hazelnut flour (positive control: 100% intensity) with the autoclaved samples: 121°C 15 mins., 121°C 30 mins., 138°C 15 mins and 138°C 30 mins. Indeed, severe allergenicity reduction *in vitro* was ensured via p ≤ 0.05 value obtained via Quantity One software and excel for all studied patients.

(d) When loading ~25 μg of soluble proteins in loading buffer, the raw hazelnut flour does not show a different protein pattern compared to the processed ones by HHP treatments (from Sera 3 to 17) (down-panel HHP Figure1 A, B, C). Nevertheless, the raw hazelnut flour shows a different protein pattern compared to that processed by AC treatments. Strongest conditions of the AC treatment decrease the IgE binding of allergenic hazelnut proteins (from Sera 3 to 17). In the case of the analysis of the Serum 1 (CAP = 0) a band slightly higher than 15 KDa is observed in the lanes of the raw flour, 121°C 15 mins., and 121°C 30 mins. Nevertheless, this protein-band (> 15KDa) disappears in the lanes of the 138°C 15 mins., and 138°C 30 mins. In the case of the analysis of the Serum 2 (CAP < 0,35): (i) The raw hazelnut flour does not show a different protein pattern compared to that processed by HHP treatments (down- panel HHP Figure1). (ii) The raw hazelnut flour shows a different protein pattern compared to that processed by AC treatments. Strongest conditions of the AC treatment decrease the IgE binding of allergenic hazelnut proteins. (up-panel AC Figure1 A, B, C).

(e) In addition, other negative control (as internal control, intra-assay) which was also used, is the incubation of raw hazelnut flour with the first and secondary antibodies (a mouse anti-human IgE mAb HE-2 ascitic fluid and a goat anti-mouse IgG peroxidise- conjugated antibody) respectively. In the lane of the negative control protein-bands were not visualized as a good evaluation.

#### CAP-FEIA of patients allergic to hazelnuts

Patients allergic to hazelnut proteins were selected for this research study.

Fifteen patients showed clinic symptoms including oral allergy syndrome, acute urticaria, systemic anaphylaxis, rhinoconjunctivitis, angioedema and avoidance (avoidance: advised to avoid hazelnut food because of a positive skin test).

The resulting data including the age and sex, are illustrated in the Table1.

### Structural analysis Modelling procedures of hazelnut proteins Cor_a_1.04, Cor_a_8, Cor_a_9 and Cor_a_11

**Figure 2:**
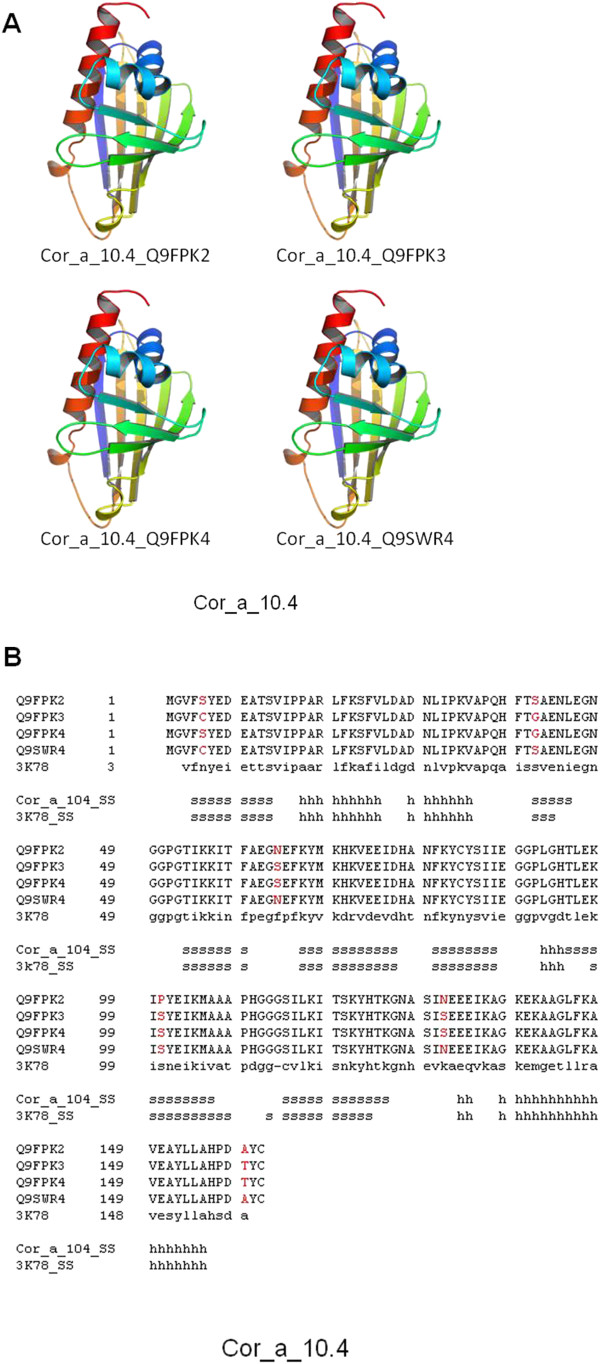
**3D structure models for Cor_a_1.04 proteins.** (**A)**. 3D models for the four Cor_a_1.04 proteins (Uniprot codes: Q9FPK2, Q9FPK3, Q9FPK4 and Q9SWR4). Models are coloured from N-ter (blue) to C-ter (red) ends. (**B)**. Structure alignment of the four Cor_a_1.04 proteins identified by their Uniprot codes, and the crystal structure of major pollen allergen Bet v 1d/h from *Betula pendula* (PDB code: 3 K78; [35]). Secondary structure elements of both the model and template are included. Differences among the sequences of the four Cor_a_1.04 proteins are indicated in red.

3D structural models were generated for the allergenic hazelnut antigens of this study:

Cor_a_1.04, Cor_a_8, Cor_a_9 and Cor_a_11 proteins. In the four cases, the structure of close homologue proteins were already available in the Protein Data Bank as crystallized structures, allowing the generation of good quality models. Cor_a_1.04 antigen is represented by four individual, closely related, proteins (Uniprot database codes: Q9FPK2, Q9FPK3, Q9FPK4 and Q9SWR4). Models for the four Cor_a_1.04 proteins were constructed using as template the crystal structure of the close relative major pollen allergen Bet v 1d/h from *Betula pendula* (PDB code: 3 K78; [37]). Due to the very similar sequence of the four proteins (Figure2B), the generated 3D models were nearly identical (Figure2A), exhibiting a complex alpha + beta scaffolding.

**Figure 3:**
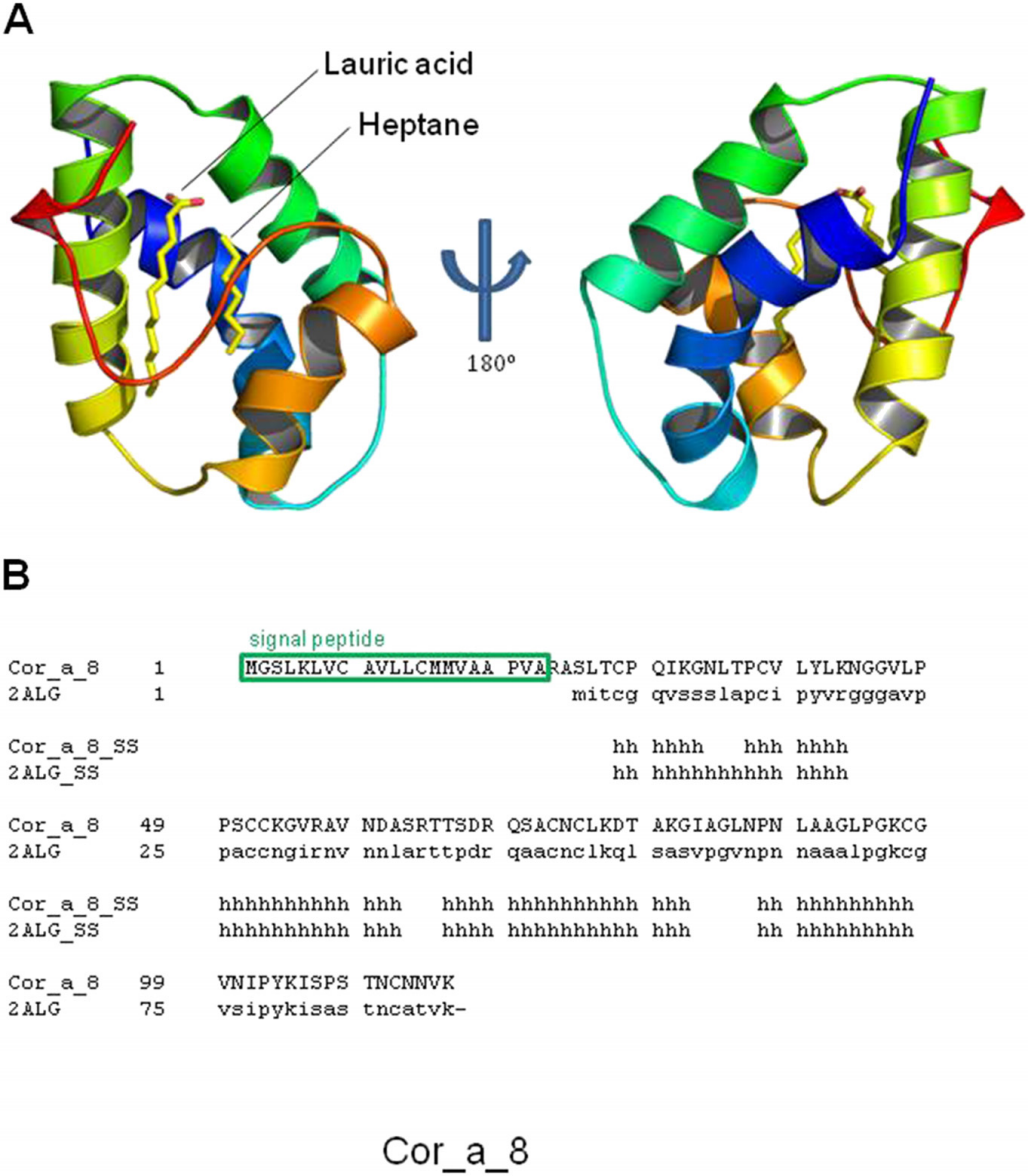
**3D structure model for Cor_a_8 protein.** (**A)**. 3D model for Cor_a_8 protein. **A.** Front (left) and reverse (right) faces are shown. Position of putative lipid molecules, present in the template structure, is indicated. (**B)**. Structure alignment of Cor_a_8 protein and the crystal structure of Pru p3, non-specific lipid transfer protein from *Prunus persica* (PDB code: 2ALG_A; [36]). Secondary elements are indicated as in Figure1. Position of signal peptide is also highlighted.

Model for the soluble domain of Cor_a_8 protein, after signal peptide processing, was built according to the 3D structure of the crystal structure of Pru p3, non-specific lipid transfer protein from *Prunus persica* (PDB code: 2ALG_A; [38]). Figure3A shows the structure of the obtained model, enriched in alpha helix elements located in close contact to two lipid molecules, as predicted according to its sequence similarity to the template (Figure3B).

**Figure 4:**
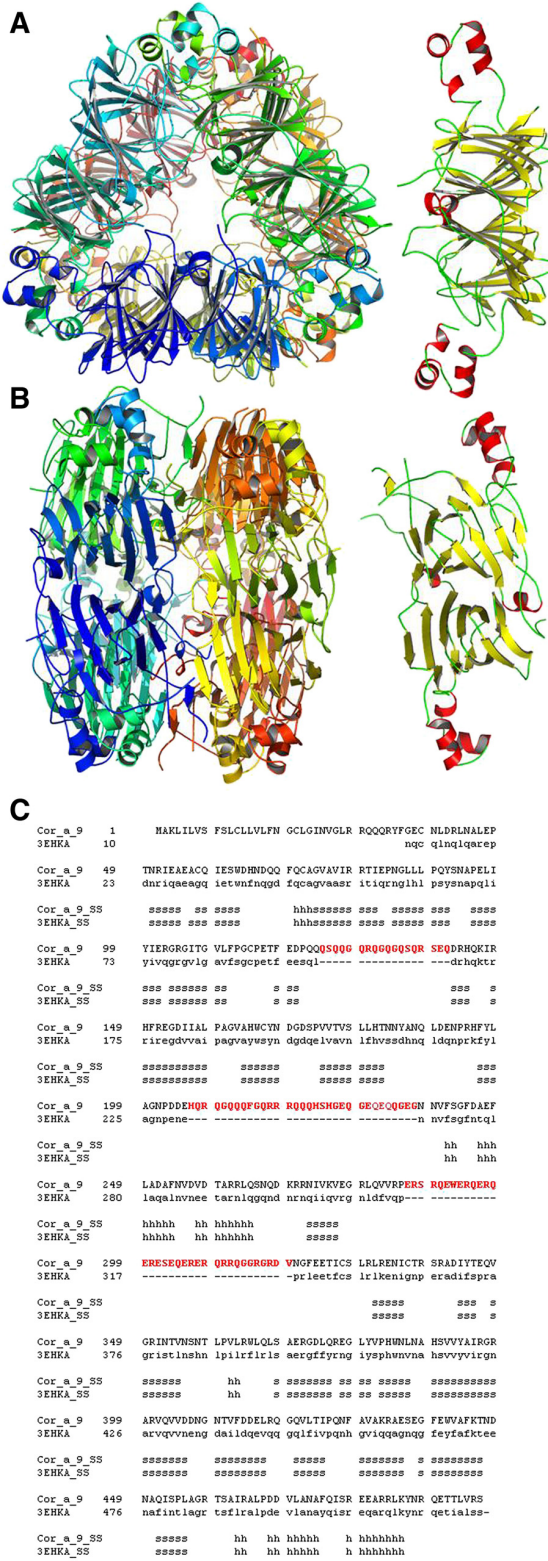
**3D structure model for Cor_a_9 homohexameric protein.** (**A)**. 3D model for Cor_a_9 hexamer (left) and single chain (right), top view. (**B)**. Same as A, side view. (**C)**. Structure alignment of Cor_a_9 protein sequence and the crystal structure of Pru du amandin from *Prunus dulcis* (PDB code: 3EHK; [37]). Secondary elements are indicated as in Figure1. Position of signal peptide is highlighted. Non-structured segments, absent in the template structure and therefore not modelled for Cor_a_9 sequence, are coloured in red.

In the case of Cor_a_9 allergen, a extremely complex, beta sheet-enriched, structure is predicted composed of six monomers arranged in two trimeric structures positioned in a base-to-base dimeric assembly (Figure4A-B), as deduced by the close sequence similarity to the crystal structure of Pru du amandin from *Prunus dulcis* (PDB code: 3EHK; [39]), as shown in Figure4C. Some protein segments, probably forming mobile non-structured loops, were not included in the model (Figure4C, residues coloured in red) due to the lack of an adequate structural template, as their counterparts are also missing in the original crystal structure.

**Figure 5:**
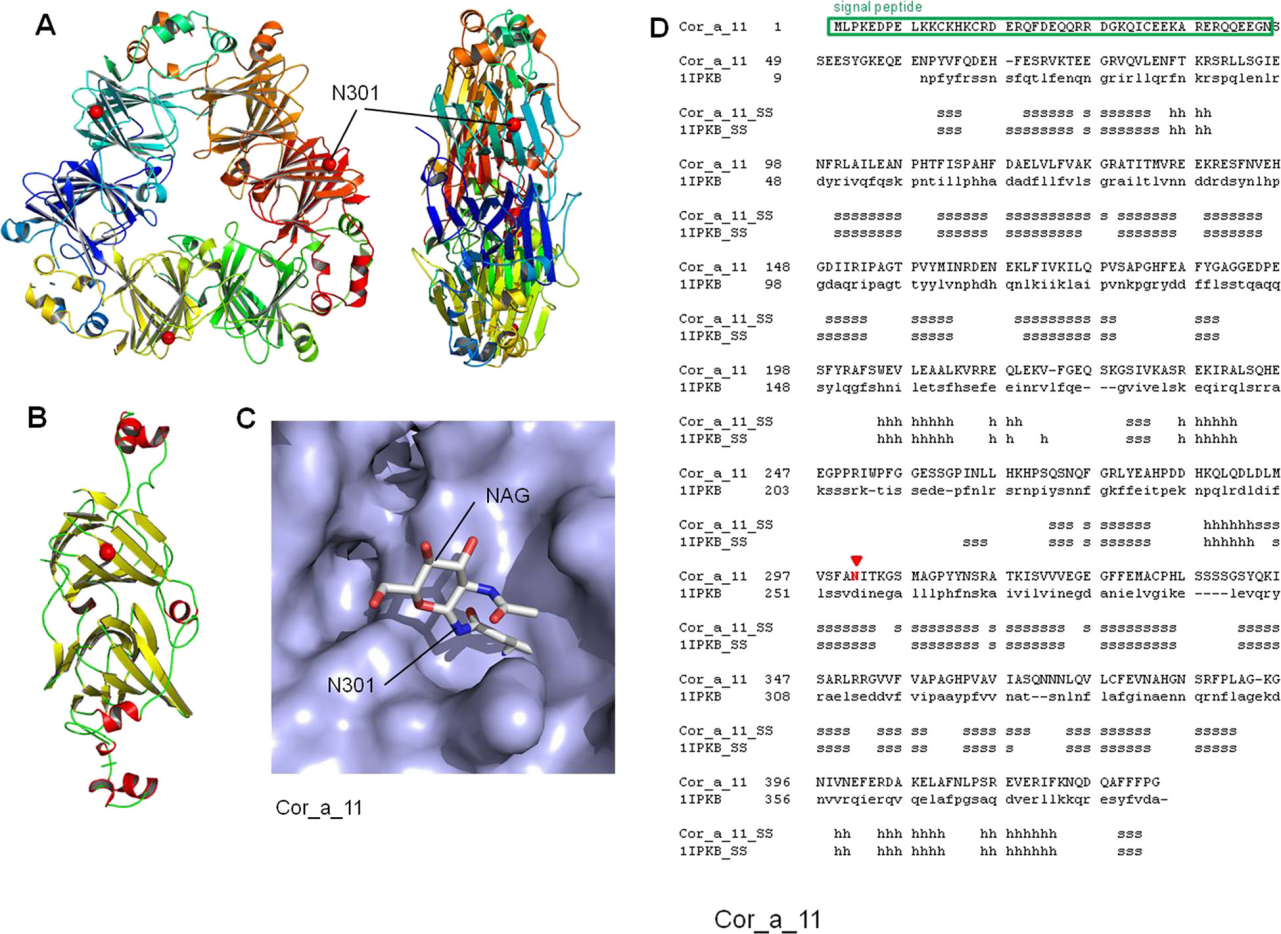
**3D structure model for Cor_a_11 homotrimeric protein. (A)**. 3D model for Cor_a_11 trimer. Front (left) and side (right) faces are shown. Position of putative glycosylated Asn301 is indicated. (**B)**. Cor_a_11 protein, single chain. (**C)**. Putative position of glycosylated Asn301 in the surface of the protein. (**D)**. Structure alignment of Cor_a_11 sequence and the crystal structure of soybean beta-conglycinin beta protein (PDB code: 1IPK; [38]). Positions of signal peptide and the putative glycosylated Asn301 residue are highlighted.

Similarly to Cor_a_9, Cor_a_11 antigen was also modelled as a trimeric structure, formed by three beta sheet-enriched monomers (Figure5A-B). Modelled spatial arrangement is equivalent to that exhibited by the selected template, the crystal structure of soybean beta- conglycinin beta protein (PDB code: 1IPK; [40]) based on their sequence similarity (Figure5D). It has been reported that Cor_a_11 was glycosylated in residue Asn301 via MALDI–TOF (matrix-assisted laser-desorption ionization–time-of-flight) MS mode analyses [41]. Position of a molecule of N-acetyl-D-glucosamine (NAG) was also modelled bound to Asn301, a residue located in a very structured, beta sheet-enriched domain. As shown -for the first time- in Figure5C, glycosylated residue is positioned on the surface of the protein, additionally validating the model.

The obtained structural models of the four studied allergenic proteins indicated in all cases that a highly complex arrangement is present in all cases, including both alpha and beta secondary elements as well as multi-domain and homo-oligomeric configurations. Recently, it has been concluded, using Synchrotron-based Fourier transform infrared microspectroscopy [30], that AC treatments substantially modified the protein structure alpha-helix to beta-sheet ratio. Taking into account the structured scaffolds of Cor_a_1.04, Cor_a_8, Cor_a_9 and Cor_a_11 pro-teins, as deduced from bioinformatics modelling, disorganization of such structures would lead to large modifications in the arrangement of their respective structure-dependant allergenic motifs.

## Discussion

The hazelnut was selected in this research study for two reasons: (1) it is well recognized that allergies to hazelnuts are tending to be of a more severe nature, causing life-threatening and unfortunately sometimes fatal reactions, and (2) it is also an edible nut with health benefits, as it is one of the best known sources for vitamin E, and a good source also for B1, B2 and B6 vitamins. Indeed, the hazelnut has several important health benefits in protection against diseases. Moreover, for example, vitamin E -found in a high level/amount in hazelnuts- prevents the factors which prepare the basis for cancer disease. The reason is because if cancer is already formed in the body, then the vitamin E contained in hazelnuts fights to defuse the harmful cells [6-14]. In addition, the patients were selected as they clearly showed clinic hazelnut allergy data (Table1).

**Figure 6:**
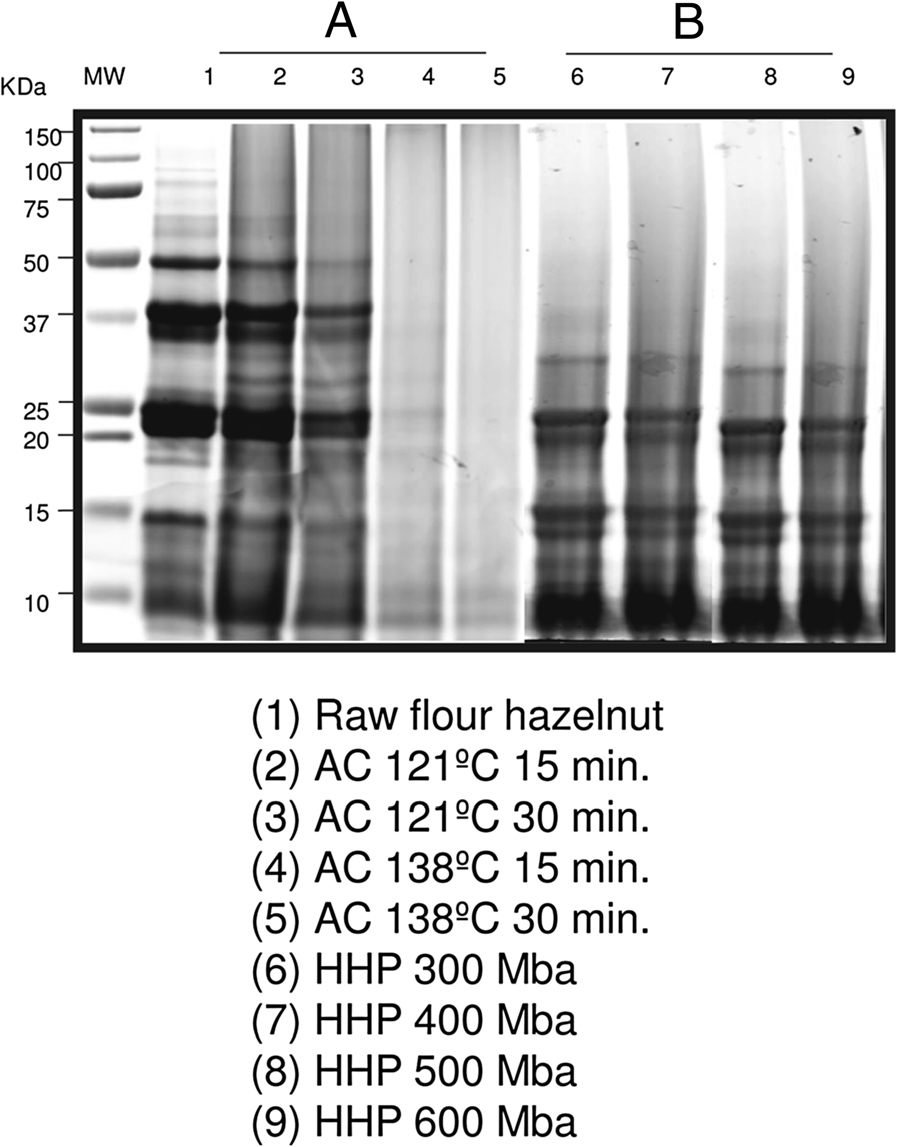
**Hazelnut flour samples analyzed by SDS-PAGE 4–20%: the wild-raw proteins and the raw-processed proteins by autoclaving and high pressure with different treatments are illustrated. (A)** The electrophoresis analysis of hazelnut flour samples shows that the different protein- patterns can be observed once the raw flour (lane 1) is processed by autoclaving (AC) treatments (lanes 2, 3, 4 and 5). Lane 2 corresponds to AC 121°C 15 mins. Lane 3 corresponds to AC 121°C 30 mins. Lane 4 corresponds to AC 138°C 15 mins. Lane 5 corresponds to AC 138°C 30 mins. ~25 μg of soluble proteins in loading buffer (containing 1 M -mercaptoethanol) was inserted into each lane. **(B)** By electrophoretic analysis of hazelnut flour samples, it is observed that the protein- patterns of the raw (lane 1) show similar patterns when processed by high pressure (HHP) treatments (lanes 6, 7, 8 and 9). Lane 6 corresponds to HHP 300Mba. Lane 7 corresponds to HHP 400Mba. Lane 8 corresponds to HHP 500Mba. Lane 9 corresponds to HHP 600Mba. ~25 μg of soluble proteins in loading buffer (containing 1 M -mercaptoethanol) was inserted into each lane. (MW: molecular weight, AC: autoclave process, HHP: high pressure process, KDa: kilodalton).

Our results show for the first time that HHP processing (300Mba, 400Mba, 500Mba and 600Mba) of hazelnut flour samples analyzed via western blotting analyses did not manifest any effects on the IgE binding of Cor_a_1, Cor_a_8, Cor_a_9 and Cor_a _11 protein- allergens in the 15 studied patients (Figure1 A, B, C). Also, the HHP hazelnut processed samples show a similar protein-pattern compared to the same samples without any HHP processing (raw hazelnut) via SDS-PAGE gel (Figure6B).

A relevant point to make, is that when observing the western-blotting analyses of serum 2 (CAP < 0,35 kilounits/L) –this serum corresponds to a patient without allergy to hazelnut- protein-bands of a molecular weight (MW) ~37 and ~15 Kda are observed (Figure1 A, Serum 2). We assume this is –maybe- because of cross-reactivity among different edible nuts. It has been described that people with hazelnut allergies can also often suffer from reactions triggered by a number of different types of nuts, even though they do not come from closely related plant species [15-18].

At this level, one can ask: Why do some individuals show cross-reactivity to homologous proteins in tree nuts? One explanation could be that while single amino acid differences are very important in individual reactivity, the visualizations of the identified IgE binding sites via 3D, can achieve relevant biological understanding about the possible relationship among structures and sequences. Moreover, if IgE binding sequences of related proteins have similar properties, the underlying assumption is that for a group of cross-reactivity allergenic proteins, the IgE epitopes areas have similar binding affinities for the same antibodies, and have, thus, common physic chemical properties in the antibody binding sites [25-29]. Furthermore, PTM analyses (*e.g.* glycosylation) allow a comprehensive investigation of complete allergens. It provides clues to elucidate mechanisms of the structures that contribute to allergenicity, which thus, in turn, help alleviate food allergens [47-50], and so improve the knowledge of immunologic disorders as allergenicity processes (see Figures 2,3,4 and 5).

AC processing (121°C 15 mins., 121°C 30 mins., 138°C 15 mins., and 138°C 30 mins.,) of hazelnut-flour samples analyzed via western blotting manifest that, the IgE binding of Cor_a_1, Cor_a_8, Cor_a_9 and Cor_a _11 protein-allergens decreases in the 15 allergic studied patients. Indeed, immunoreactive hazelnut protein-bands disappear (Figure1 A, B, and C, serum num. 3 until serum num. 17). In addition, via SDS-PAGE analysis, we could observe that the protein-patterns from the AC hazelnut-processed samples were different compared to the protein-pattern of the raw-hazelnut (without AC treatment) (Figure6 A). It would seem relevant to ask at this point: are protein-allergens from hazelnut-processed samples via AC processing aggregated? Without much doubt, yes, and probably PTMs (*e.g.* glycosylation) are altering the molecular structure of some hazelnut allergen-proteins when they are autoclaved [15-20,42-51]. Thus, AC processing of hazelnut seems to be affecting the 3D-structure of its allergen-proteins. Perhaps due to the AC-processing, it might possible that Ag-Ab-interactions are modified, as hazelnut IgE mediated allergy is triggered by the proteins which resist processes such as cooking –for example [52-59].

In the four modelled antigen proteins, with the exception of the all-alpha Cor_a_8 protein, 3D structure can be classified as a mixed alpha + beta scaffolding, including also a variable percentage of non-structured loops connecting the secondary structure elements. From the point of view of antigenic properties, epitopes can be divided in linear and structural ones, corresponding mainly to sequence regions folded into well defined 3D structure (alpha and beta chains) or into mainly unstructured loops. As has been demonstrated previously [30], AC treatments modified the alpha to beta ratio, thus affecting mainly the 3D epitopes, and to a lesser extent to linear epitopes potentially located among them. Structure models for Cor_a_1.04 proteins (Figure2) showed that most of the protein sequence is struct ured, the loops being located almost exclusively as the apical structures of beta-turns, and so very influenced by the beta structure itself. In this case, AC treatments are expected to be very effective, as mainly all epitopes are predicted to be structure-dependant.

The model for the soluble domain of Cor_a_8 protein (Figure3) exhibited a very similar arrangement, the small-size loops being located exclusively as linkers between alpha helices. Equally as for Cor_a_1.04 proteins, AC treatment for Cor_a_8 is predicted to disorganize almost all possible epitopes. The large and complex model for Cor_a_9 antigen (Figure4), is, in contrast, predicted to be composed not only of a very structured, beta-sheet enriched, protein core, but also of long unstructured loops. These loops (red sequences in Figure4C) cannot be modelled due precisely to their lack of stable structure, but they are predicted to carry linear epitopes, as they are located in the external faces of the protein hexamer and thus exposed to the solvent.

Nevertheless, no band of a compatible size was detected after lengthy AC treatments (Figures 1 and 6), probably indicating that the predominant antigenicity was due to structural and not linear epitopes. Finally, model for Cor_a_11 protein (Figure5) indicated that this protein contains a low proportion of unstructured loops, being enriched in beta-sheet structures. According to this fact, AC treatment resulted in the lack of antigenicity. The model of Cor_a_11 also predicted a suitable structure arrangement for a NAG group attached to Asn 301 residue, as the initial site for glycosylation of the protein. Position of Asn 301 in a structured beta sheet makes this position a candidate for structural changes after AC treatment and thus to a putative loss of antigenicity due to the presence of sugar moiety. In addition, *in vivo* assays with the AC-processed hazelnut samples (for example via Prick- Prick) will be performed in the future, in order to verify if specific immunoreactions, mediated by IgE, decrease in those patients diagnosed with allergy to hazelnuts [60,61].

## Conclusions

An important reduction in allergenicity *in vitro* to hazelnut flour after AC processing was observed in the allergic clinic patients studied via western blotting analyses, while no reduction in allergenicity after HHP processing was observed.

Allergenicity processes are in a great manner dependent on the molecular recognition of specific structure motifs of proteins and their variation could be a putative explanation for the effects of AC processing on the allergenicity of the immunoreactive hazelnut protein-bands. Thus, a series of homology-based bioinformatics 3D models were generated. The visualization of a relevant glycosylation for the first time in the protein-allergen Cor-a-11 structure was observed, showing a new role which could open a new door for allergenicity- unravellings according to the data presented in this article.

A high number of technique-combination-approaches are available for clinical allergy research. It is always necessary to test different strategies in order to reach a greater level of efficiency for your clinical study according to the type of samples to be analyzed. We aimed to detail the current and useful techniques to carry out *in vitro* allergenic studies which may be helpful for understanding hazelnut allergy, IgE mediated. This study is also aimed at combining functional research protein-allergen analysis with structural analysis of allergenic proteins, in order to visualize putative PTMs as glycosylation, which can help to unravel biological understanding with clinical significance. Nowadays it is essential that clinicians and scientific experts work together in order to improve the therapies and diagnosis advances for hazelnut allergy disease.

## Competing interests

The authors declare that they have no competing interests.

## Authors’ contributions

EL carried out the immunoblotting analyses with human sera, and structural and modelling studies of hazelnut proteins Cor_a_1.04, Cor_a_8, Cor_a_9 and Cor_a_11 (Centro de Investigación (i + 12) del Hospital Universitario 12 de Octubre, Avda de Córdoba s/n 28041, Madrid, Spain). CC and CB provided the AC and HHP processing samples and the SDS_PAGE gel (Departamento de Tecnología de Alimentos, SGIT-INIA, Ctra. La Coruña Km 7.5, 28040 Madrid, Spain). MAJ, JR and JFC provided the patient sera (Hospital Universitario 12 de Octubre, Servicio de Alergia, and Centro de Investigación (i + 12) Av. de Córdoba s/n 28040, Madrid, Spain). All authors revised it critically for intellectual content and publish this article. All authors read and approved the final manuscript.
